# The three-year incidence of major hemorrhage among older adults initiating chronic dialysis

**DOI:** 10.1186/s40697-014-0021-x

**Published:** 2014-09-02

**Authors:** Manish M Sood, Sarah E Bota, Eric McArthur, Moira K Kapral, Navdeep Tangri, Greg Knoll, Deborah Zimmerman, Amit X Garg

**Affiliations:** Ottawa Hospital Research Institute, Department of Medicine, University of Ottawa, Ottawa Institute for Clinical Evaluative Sciences, Civic campus, 2-014 Administrative Services Building, 1053 Carling Avenue, Box 693, Ottawa, ON Canada; Institute for Clinical Evaluative Sciences, Toronto, ON Canada; Department of Epidemiology and Biostatistics, Western University, London, ON Canada; Department of Medicine, University of Toronto, Toronto, ON Canada; The Institute of Health Policy Management and Evaluation, University of Toronto, Toronto, ON Canada; Seven Oaks General Hospital, Winnipeg, MB, Ontario Canada; Kidney Clinical Research Unit, Division of Nephrology, Western University, London, ON Canada; Division of Nephrology, Department of Medicine, Western University, London, ON Canada

## Abstract

**Background:**

For those who initiate chronic dialysis, knowing what proportion will experience 3-year outcomes of hemorrhage with hospitalization informs patient prognosis, disease impact, and the planning of trials and programs to prevent events.

**Objectives:**

We examined the incidence of hemorrhage and related gastrointestinal endoscopic procedures in incident older dialysis patients and stratified patients by age, era, dialysis modality and whether recently prescribed anti-thrombotic medication.

**Design:**

Retrospective cohort study

**Setting:**

Ontario, Canada from 1998 to 2008 (n = 11,173)

**Patients:**

All older patients (>65 years) who initiated chronic dialysis

**Measurements:**

Hospitalization with hemorrhage and its subtypes (upper and lower gastrointestinal, intra-cerebral, subarachnoid) and related-gastrointestinal procedures.

**Methods:**

Three-year outcomes of hospitalization with hemorrhage were expressed as cumulative incidence and incidence rate (number of events per 1,000 patient years). Results were stratified by patient age (66 to 74, 75 to 84, ≥ 85), era (1998 to 2001, 2002 to 2005, 2006 to 2008) and dialysis modality. Among those with hemorrhage, we examined prescriptions for anti-thrombotic medications (warfarin, clopidogrel) in the preceding 120 days.

**Results:**

The 3-year cumulative incidence of hemorrhage was 14.4% (roughly 1 in 7 patients). By location, the 3-year cumulative incidence was 8.9% lower gastrointestinal, 6.1% upper gastrointestinal, 0.9% intra-cerebral and 0.1% sub arachnoid hemorrhage. The 3-year cumulative incidence of gastrointestinal endoscopic procedures was 14.8%. The cumulative incidence and rate of hemorrhage were not appreciably different across the 3 age strata, by era or by dialysis modality. Among patients with a hemorrhage, 29.5% were prescribed warfarin in the preceding 120 days, and 8.4% clopidogrel.

**Limitations:**

Recurrent events were not included.

**Conclusions:**

Many older patients who initiate chronic dialysis will be hospitalized with hemorrhage and receive related procedures over the subsequent three years. Despite greater age and co-morbidity over the last decade this incidence has not changed.

**Electronic supplementary material:**

The online version of this article (doi:10.1186/s40697-014-0021-x) contains supplementary material, which is available to authorized users.

## Abrégé

**Contexte:**

Dans les cas de dialyse chronique, connaître la proportion de patients qui souffriront d’hémorragies nécessitant une hospitalisation dans les trois années suivantes fournit un éclaircissement sur le pronostic des patients, les répercussions de la maladie et la planification des essais et des programmes visant à prévenir ces épisodes.

**Objectifs:**

Nous avons examiné l’incidence des hémorragies et des procédures gastro-intestinales endoscopiques connexes chez les patients âgés débutant la dialyse, et les avons stratifiés par âge, par période, par modalité de dialyse et selon qu’on leur a récemment prescrit des médicaments antithrombotiques.

**Type d’étude:**

Étude rétrospective de cohorte

**Contexte:**

Ontario, Canada, entre 1998 et 2008 (n = 11 173)

**Participants:**

Tous les patients âgés (>65 ans) qui ont entamé une dialyse chronique

**Mesures:**

L’hospitalisation avec hémorragie et sous-types (hémorragie digestive haute ou basse, intracérébrale, sous-arachnoïdienne), de même que les procédures gastro-intestinales connexes.

**Méthodes:**

Les cas d’hémorragies nécessitant l’hospitalisation dans les trois années suivantes sont exprimés en taux d’incidence cumulatif et en taux d’incidence (nombre de cas pour 1 000 patients par année). Les résultats ont été stratifiés par âge du patient (66 à 74, 75 à 84, ≥ 85), par période (1998 à 2001, 2002 à 2005, 2006 à 2008) et par modalités de dialyse. Parmi ceux qui ont souffert d’hémorragie, nous avons examiné les prescriptions de médicaments antithrombotiques (warfarine, clopidogrel) au cours des 120 jours précédents.

**Résultats:**

Le taux d’incidence cumulatif des hémorragies sur trois ans était de 14,4% (environ 1 patient sur 7). Par localisation du saignement, le taux d’incidence cumulatif sur trois ans indiquait une hémorragie digestive basse à 8,9%, intracérébrale à 0,9% et sous-arachnoïdienne à 0,1%. Le taux d’incidence cumulatif des procédures gastro-intestinales endoscopiques était de 14,8%. Le taux d’incidence cumulatif et le taux d’incidence des hémorragies ne variaient pas sensiblement en fonction de l’âge, de la période ou de la modalité de dialyse. Parmi les patients ayant souffert d’hémorragie, 29,5% ont reçu une prescription de warfarine au cours des 120 jours précédents, et 8,4%, de clopidorel.

**Limites de l’étude:**

Les cas récurrents n’ont pas été considérés.

**Conclusion:**

Plusieurs patients âgés qui entament une dialyse chronique seront hospitalisés pour hémorragie et subiront les procédures connexes au cours des trois années suivantes. Malgré un âge plus avancé et une comorbidité au cours de la dernière décennie, la fréquence n’a pas changé.

## What was known before

Dialysis patients are known to experience a significant risk of hemorrhagic events. Accurate determination of the risk has been difficult due to variations in the definition of hemorrhage and the use of non-validated diagnostic codes in previous studies. Further it remains unclear if or to what magnitude the hemorrhagic risk changes with increasing age and over time.

## What this adds

We found that one in seven patients (14.43%) will experience a hospitalization with a hemorrhage within the first three years of initiating dialysis and this high risk did not increase with age or improve over time.

## Background

Patients who initiate chronic dialysis are older, have more co-morbid illness than a decade prior, and subsequently may experience more hospitalizations in follow-up. Hemorrhages are one of most common hospital admission diagnoses in the end-stage renal disease population and contribute significant morbidity and mortality [1]. Compared to the general population, the incidence of hemorrhage is considerably higher in those receiving dialysis with rates ranging from 35.4 to 80 per 1000-patient years (which is contingent on the study design, the definition used, method of outcome ascertainment and concurrent use of anti-thrombotic medications such as clopidogrel or warfarin) [2,3].

In the non-dialysis population, the incidence of hemorrhagic events has been declining over the last two decades, attributed to the widespread use of gastrointestinal endoscopic procedures, treatments for *H.pylori*-related ulcers and the use of proton pump inhibitors [4–8]. In an American dialysis population, the incidence of non-variceal upper gastrointestinal bleeding had not changed over the last decade despite increasing patient comorbidity [9,10]. It is unknown whether the same is true in other jurisdictions, or applies to other types of bleeding (lower gastrointestinal, intracranial bleeding) or related procedures (gastrointestinal endoscopy). Furthermore, the risk of bleeding appears to increase with advancing age [7]. In the non-CKD, non-dialysis population, hospitalizations for UGI hemorrhage per 100, 000 population increased dramatically with age (<65 31.7, 66 to 75 197.4, > 75 425.2) with a similar trend observed for lower GI hemorrhage. As the average age of incident dialysis patients is increasing over time, it follows that there may a parallel rise in the risk of major hemorrhage [11]. Lastly, the hemorrhagic risk may differ based on the dialysis modality as patients on peritoneal and hemodialysis are well known to differ based on co-morbidities, exposures (to heparin with the hemodialysis procedure) and functional status.

In this study we examined older adults who initiated chronic dialysis in Ontario, Canada over a ten-year period to determine the 3-year cumulative incidence and incidence rate (per 1000 person years) of hemorrhage and related gastrointestinal endoscopic procedures. We further examined hemorrhage incidence across three age (66 to 74, 75 to 84, ≥85), era (1998 to 2001, 2002 to 2005, 2006 to 2008) and dialysis modality (peritoneal, hemodialysis) and considered whether patients with hemorrhagic events were recently prescribed anti-thrombotic medication.

## Methods

### Study design and setting

We conducted a population-based study of all older adults who initiated chronic dialysis in Ontario, Canada between January 1, 1998 and December 31, 2008 using linked health care databases. Ontario has approximately 13 million residents, 14% of whom are 65 years of age or older [12]. Residents have universal access to hospital care and physician services and those 65 years of age or older have universal prescription drug coverage. All healthcare encounters are entered in administrative databases and are held in a linked, de-identified form at the Institute for Clinical Evaluative Sciences (ICES). We conducted this study according to a pre-specified protocol that was approved by the research ethics board at Sunnybrook Health Sciences Centre (Toronto, Canada). The reporting of this study followed guidelines for observational studies [13] (Additional file 1: Table S1). Regional ethics approval was obtained.

### Data sources

We ascertained patient characteristics, drug use, covariate information, and outcome data using records from five linked databases. We identified patients initiating chronic dialysis in Ontario through the Canadian Organ Replacement Registry (CORR) [11]. CORR is a national dialysis registry with information collected from all dialysis facilities in Canada on incident chronic dialysis patients [14,15]. The recorded information includes dialysis modality and the type of vascular access. We obtained vital statistics from the Ontario Registered Persons Database which contains demographic information on all Ontario residents who have ever been issued a health card. We used the Ontario Drug Benefit Plan database to identify prescription drug use. This database contains highly accurate records of all outpatient prescriptions dispensed to patients aged 65 or older, with an error rate of less than 1% [16]. We identified diagnostic and procedural information on all hospitalizations from the Canadian Institute for Health Information Discharge Abstract Database (CIHI-DAD). We obtained information from the Ontario Health Insurance Plan database, which includes health claims for inpatient and outpatient physician services. Previously, we have used these databases to research renal health outcomes and health services [17–19]. Whenever possible, we defined patient characteristics, dialysis characteristics and outcomes using database codes that have been proven reliable when compared with manual chart review (Additional file 1: Table S2).

### Patients

We studied patients who initiated chronic dialysis therapy within our 10 year accrual period. CORR captures patients who require chronic dialysis therapy; their date of dialysis initiation served as the index date (also referred to as the cohort entry date). We excluded the following people from analysis: i) those without at least one year of eligibility for prescription drug coverage (age less than 66) to avoid incomplete medication records, ii) those with a prior history of kidney transplantation or prior chronic dialysis, to focus on incident end-stage renal disease treated with chronic dialysis and iii) those who initiated chronic dialysis during a hospital stay and died prior to hospital discharge.

Known demographics and co-morbid risk factors for hemorrhage were recorded including age, hypertension, prior major hemorrhage [20], diabetes, cardiovascular disease including prior myocardial infarction, and atrial fibrillation in the five years preceding dialysis initiation. Antithrombotic therapy (warfarin, clopidogrel) was captured in the 30 and 120 days preceding the hemorrhagic event. In Ontario, acetyl-salicylic acid (ASA) is often available without a prescription and thus would not be captured in the Ontario Drug Benefit Plan.

### Outcomes

All individuals were followed for three years after the initiation of chronic dialysis for the outcomes of interest. The primary outcome was the first hospitalization with non-traumatic major hemorrhage, a composite outcome defined by any of the following: upper and lower gastrointestinal (GI) hemorrhage, intra-cerebral hemorrhage (ICH) and subarachnoid hemorrhage (SAH). The secondary outcome was each specific subtype of hemorrhage and all gastrointestinal endoscopic procedures that occurred during the hospital stay.

The components of non-traumatic major hemorrhage were specifically selected based on prior validation studies and the severity of each event [21,22]. Previous studies have reported positive predictive values of 90-98% for each of the specific subtypes of hemorrhage we used as secondary outcomes [21,22]. Non-validated hemorrhages, such as those associated with vascular access, and non-severe hemorrhages (e.g. epistaxis) were excluded. Gastrointestinal endoscopic procedures included gastroscopy, colonoscopy, sigmoidoscopy, duodenoscopy, & esophagoscopy. Hospitalizations for outcomes were identified using the International Classification for Disease (ICD), 9 and 10 codes (Additional file 1: Table S3).

### Statistical analysis

The study period was categorized into three eras: 1998–2001, 2002–2005, 2006–2008, three age groups: 66–74, 75–84 and ≥85 years of age and dialysis modality (peritoneal dialysis and hemodialysis). Differences between baseline characteristics were evaluated using analysis of variance (ANOVA) and chi-squared tests. We determined frequencies and calculated a modified Charlson comorbidity index for all patients at baseline by era [23].

We calculated the three-year cumulative incidence of hemorrhagic events (defined as the proportion of patients who experienced the event at least once within the three-years of follow-up in percent) and three-year incidence rates (defined as the rate per 1,000 person-years of follow-up). For the primary outcome, patients were censored at the time of their first hemorrhagic event, death or end of follow-up period whereas for the secondary outcome of subtypes of hemorrhage or related-procedures, patients were censored only after the specific subtype of hemorrhage or related-procedure occurred, death or the end of the follow-up period. This allowed us to determine the specific subtype or related procedure incidence. Primary results were stratified by era (1998 to 2001, 2002 to 2005, and 2006 to 2008), age category (65–74, 75–84, ≥85) and dialysis modality. Trends were examined using the Cochrane-Armitage test. We examined whether patients with hemorrhage were prescribed anti-thrombotic medications (warfarin, clopidogrel) in the 30 days and 120 days preceding their hemorrhagic event. Of note, the three-year cumulative incidence does not account for time at risk, representing simply a proportion of events achieved with 3 years. In this regard, the incidence rate was also calculated.

## Results

Our final study cohort included 11, 173 incident chronic dialysis patients comprising 19,894.2 patient-years of follow-up with the average follow-up per person of 677 days (see cohort development Additional file 1: Figure S1). Baseline characteristics by era and age are presented in Table 1. Most patients were Caucasian (versus other races) and initiated hemodialysis (>80% of the cohort, versus peritoneal dialysis). A history of atrial fibrillation and hemorrhage were common in the older age groups. Patients who initiated dialysis in more recent years (versus earlier years) were slightly older and had greater comorbidity. Prescriptions at dialysis initiation for warfarin were unchanged (mean 12.2%, p = 0.098) whereas clopidogrel significantly increased from 0.5 to 8.0% (p < 0.001) in the more recent era compared to earlier. Among co-morbidities prior to dialysis initiation, hemorrhage, acute coronary syndrome and the presence of a mechanical heart valve were more common in the recent era.

**Table 1:** **Baseline characteristics of patients initiating chronic dialysis in Ontario, Canada from 1998 to 2008**

		**Era, year of dialysis initiation**				**Patient age, years**			
**Characteristics**	**Total**	**1998-2001**	**2002-2005**	**2006-2008**	**p-value**	**65-74**	**75-84**	**≥85**	**p-value**
	n = 11,173	n = 3,837	n = 4,231	n = 3,105					
**Age, mean ± SD**	75.8 ± 6.2	74.8 ± 5.8	76.1 ± 6.2	76.5 ± 6.43	<0.001				
**Women,%**	42.3	42.3	42.0	42.6	0.873	41.6	42.5	44.2	0.286
**Race,%**									
**Caucasian**	77.0	70.0	80.7	80.7	<0.001	73.9	79.4	80.5	<0.001
**East Asian**	6.2	6.6	6.1	5.8		5.9	6.2	7.6	
**South Asian**	3.0	2.6	3.1	3.5		4.1	2.4	0.7	
**Aboriginal**	1.1	1.1	1.0	1.2		1.7	0.6	0.4	
**Other**	4.9	4.4	5.1	5.1		6.2	3.8	3.5	
**Unknown**	7.9	15.5	4.0	3.6		8.1	7.7	7.4	
**Modality,%**									
**Hemodialysis**	81.4	78.5	82.3	80.4	<0.001	77.6	82.8	83.4	<0.001
**Peritoneal dialysis**	19.5	21.5	17.7	19.6		22.4	17.2	16.6	
Medication*,%									
**Clopidogrel**	4.5	0.5	5.6	8.0	<0.001	4.3	4.7	4.8	0.571
**Warfarin**	12.2	11.8	11.9	13.3	0.098	10.6	13.5	13.9	<0.001
**Co-morbidity,%**									
**Atrial fibrillation**	14.7	14.0	15.3	14.9	0.258	12.2	16.6	17.8	<0.001
**Stroke**	5.0	5.8	5.0	4.0	0.003	5.2	4.9	4.5	0.514
**Prior Major hemorrhage**	10.1	8.9	10.1	11.7	<0.001	10.0	9.8	12.4	0.039
**Acute coronary syndrome**	13.6	12.7	14.7	13.2	0.026	1.8	2.1	1.8	0.588
**Deep vein thrombosis & pulmonary embolism**	1.9	1.8	1.8	2.3	0.202	1.8	2.1	1.8	0.588
**Mechanical heart valve**	1.3	0.9	1.4	1.5	0.022	1.5	1.2	0.3	0.009
**Modified Charlson Co-morbidity Indexǂ,%**									
**2**	27.4	27.7	26.7	28.0	<0.001	26.8	27.5	30.2	<0.001
**3**	13.9	17.5	12.9	10.9		12.2	14.7	18.2	
**4**	19.0	20.1	17.3	19.9		18.6	19.0	20.9	
**≥5**	39.7	34.7	43.0	41.2		42.4	38.8	30.6	

*Medication was based on evidence of medication prescription at dialysis initiation.

ǂ We used a modified Charlson Index that gave each individual on dialysis an automatic two-points for moderate-to-severe kidney disease.

### Three-year cumulative incidence and event rate of hemorrhage

The three-year cumulative incidence of hemorrhage was 14.43% with an event rate of 52.64 per 1000 person years (see Table 2). By site of bleeding, the cumulative incidence and event rates were highest for lower gastrointestinal (8.88% and 31.21 per 1000 person years) followed by upper gastrointestinal (6.06% and 20.94 per 1000 person years), intra-cerebral (0.85% and 2.85 per 1000 person years) and subarachnoid (0.10% and 0.33 per 1000 person years). The three-year cumulative incidence and event rate for the receipt of gastrointestinal endoscopy (upper, lower) was 14.86% and 54.45 per 1000 person years of patients. Patients with hemorrhage at more than one anatomical location occurred in 162 cases (1.45%).

**Table 2:** **3-year cumulative incidence and incidence rates per 1,000 person year of total hemorrhages and hemorrhage subtypes**

	**Total hemorrhage** ^**1**^	**Lower gastrointestinal hemorrhage** ^**2**^	**Upper gastrointestinal hemorrhage** ^**2**^	**Intra-cerebral hemorrhage** ^**2**^	**Subarachnoid hemorrhage** ^**2**^	**Gastrointestinal endoscopy** ^**3**^
**N = 11, 173**	1612	992	677	95	11	1660
**3-year cumulative incidence (%)**	14.43	8.88	6.06	0.85	0.10	14.86
**Event rate (per 1000 patient-years)**	52.64	31.21	20.94	2.85	0.33	54.45

^1^Counted as the first major hemorrhage at any anatomical location.

^2^Counted as the first hemorrhage at this anatomical location.

^3^Gastrointestinal endoscopy included gastroscopy, colonoscopy, sigmoidoscopy, duodenoscopy & esophagoscopy, when the procedure occurred during hospital stay.

### Age and the three-year cumulative incidence and event rate of hemorrhage

**Figure 1:**
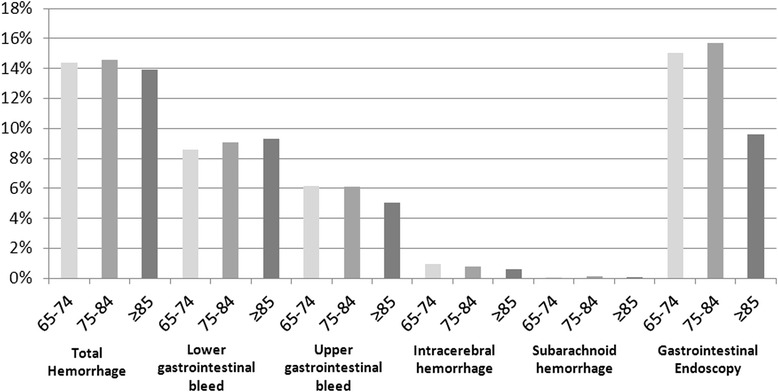
**3-year cumulative incidence rate of all hemorrhage, its subtypes and related-endoscopy, stratified by age groups.** A p-value of 0.005 was found in the gastrointestinal endoscopy group across age using Cochrane-Armitage test for trend.

The 3-year cumulative incidence and event rate for hemorrhage was similar across age groups (see Figure 1). They were highest in those 75–84 years of age (14.55%, 53.23 per 1000 person years) and lowest in those ≥ 85 years of age (13.95%, 50.78 per 1000 person years). Individuals ≥ 85 years of age had the highest 3-year cumulative incidence of lower gastrointestinal hemorrhage (9.30%, 32.85 per 1000 person years) and lowest incidence of upper gastrointestinal hemorrhage (5.04%, 17.3 per 1000 person years) and intra-cerebral hemorrhage (0.58%, 1.94 per 1000 person years). Individuals’ ≥ 85 years of age underwent substantially fewer gastrointestinal endoscopic procedures (9.59%, 34.14 per 1000 person years, p = 0.005) than those in the other 2 age categories.

### Secular trends in the rates of hemorrhage

The 3-year cumulative incidence rates and event rates (per 1000 person years) of hemorrhage were consistent across time periods and varied from 14.15 to 14.36% and 51.57 to 52.29 (see Table 3). The highest 3-year cumulative incidence and event rate for lower gastrointestinal hemorrhage was seen in 2002–2005 (9.08%, 31.97 per 1000 patient-years). In the same era, the 3-year cumulative incidence and event rate for upper gastrointestinal hemorrhage was lowest (5.91%, 20.41 per 1000 patient-years). The 3-year cumulative incidence of gastrointestinal endoscopic procedures ranged from 15.66 to 14.94% and was steady over time (p = 0.34). Intra-cerebral hemorrhages increased from 0.70 to 0.97%, p = 0.23 whereas the 3 year cumulative incidence of subarachnoid hemorrhage was relatively low.

**Table 3:** **Secular trends of the 3-year cumulative incidence (%) and event rates of hemorrhage (per 1000 person years), its subtypes, related endoscopy and all-cause mortality**

**Outcome**		**Total**	**1998-2001**	**2002-2005**	**2006-2008**	**P value**
**Total hemorrhage**	3-yr incidence	14.43	14.15	14.72	14.36	0.78
	Event rate	52.64	51.57	53.87	52.29	
**Upper gastrointestinal**	3-yr incidence	6.06	6.12	5.91	6.18	0.95
	Event rate	20.94	21.19	20.41	21.33	
**Lower gastrointestinal**	3-yr incidence	8.88	8.84	9.08	8.66	0.83
	Event rate	31.21	31.01	31.97	30.42	
**Intra-cerebral**	3-yr incidence	0.85	0.70	0.90	0.97	0.23
	Event rate	2.85	2.35	3.01	3.24	
**Subarachnoid**	3-yr incidence	0.10	0.05	0.14	0.10	0.51
	Event rate	0.33	0.17	0.47	0.32	
**Gastrointestinal endoscopy**	3-yr incidence	14.86	15.66	14.06	14.94	0.34
	Event rate	54.45	57.76	51.36	54.61	

### Dialysis modality and three-year cumulative incidence and event rate of hemorrhage

The three-year cumulative incidence and event rate for total hemorrhage and its subtypes were all lower among peritoneal dialysis patients compared to hemodialysis however the differences were not statistically significant (see Table 4). Patients on peritoneal dialysis did undergo more gastrointestinal endoscopic procedures than patients on hemodialysis (17.84 vs. 14.13%, p < 0.0001).

**Table 4:** **Total 3-year cumulative incidence and event rates of hemorrhage (per 1,000 person years), its subtypes, and related endoscopy stratified by dialysis modality**

**Outcome**		**Total**		**P value**
		**Hemodialysis**	**Peritoneal dialysis**	
**Total hemorrhage**	3-yr incidence	14.63%	13.58%	0.21
	Event rate	53.37	48.86	
**Upper gastrointestinal**	3-yr incidence	6.09%	5.92%	0.76
	Event rate	21.07	20.38	
**Lower gastrointestinal**	3-yr incidence	9.04%	8.21%	0.22
	Event rate	31.85	28.59	
**Intra-cerebral**	3-yr incidence	0.88%	0.73%	0.51
	Event rate	2.94	2.45	
**Subarachnoid**	3-yr incidence	-	-	
	Event rate	0.30	0.46	
**Gastrointestinal endoscopy**	3-yr incidence	14.13%	17.84%	<0.0001
	Event rate	51.64	66.24	

Note: 3-year cumulative incidence of subarachnoid hemorrhage cell values were suppressed for the purposes of privacy and confidentiality.

### 3-year cumulative incidence and secular trends of hemorrhage and preceding prescription for anti-thrombotic medications

Among the 1,612 patients with hemorrhages, 4.20 and 8.37 were prescribed clopidogrel in the preceding 30 and 120 days (Table 5). Warfarin prescriptions occurred in 16.6 and 29.53% in the preceding 30 and 120 days. Just over half the hemorrhagic events observed occurred within 30 days of prescription of either drug. Clopidrogrel prescription prior to major bleeding increased over time from 1998–2001 to 2006–2008 (p < 0.001) and during the same time period, warfarin prescription declined 120 days prior to the event (p = 0.023). Co-prescription of both warfarin and clopidogrel in the period prior to a hemorrhage was relatively rare with a non-significant increase over time from 0.7% to 3.4% (p = 0.07).

**Table 5:** **Secular trends of the proportion of patients who received an anti-thrombotic medication prescription 30 and 120 days preceding a hemorrhagic event**

**Medication**	**Total**	**1998-2001**	**2002-2005**	**2006-2008**	**P value**
**Clopidogrel (30 days)**	4.2%	1.7%	4.3%	7.0%	<0.001
**Clopidogrel (120 days)**	8.4%	2.6%	9.3%	14.1%	<0.001
**Warfarin (30 days)**	16.6%	17.5%	17.5%	14.3%	0.316
**Warfarin (120 days)**	29.5%	33.3%	29.2%	25.3%	0.023
**Warfarin + Clopidogrel (120 days)**	1.8%	0.7%	1.6%	3.4%	0.072

## Discussion

In this population-level study of older incident dialysis patients in Ontario, Canada, the 3-year cumulative incidence of hospitalization with hemorrhage was 14.43% or one in seven patients. The most common site of bleeding was lower gastrointestinal followed by upper gastrointestinal, intra-cerebral and subarachnoid hemorrhage, and many patients received an endoscopic procedure during their hospital admission. The incidence of hemorrhage was stable across age groups, over time, by dialysis modality and elevated among those recently prescribed anti-thrombotic medications. Among incident dialysis patients, the burden of hemorrhage is high and population-specific interventions are warranted.

Our finding of a high incidence of hemorrhage among incident dialysis patients with an observed rate of 52.64 per 1,000 person years is consistent with previous studies [2,3,24]. In prior large dialysis cohort studies event rates have ranged from 35.4 to 80 per 1,000 person-years [2,3]. Differences in the reported rates are attributable to variations in the definition of hemorrhage and inclusion criteria of individual studies. Olsen *et al.* examined hemorrhage requiring hospitalization in patients with atrial fibrillation and included urinary tract and airway hemorrhage [3]. Sood *et al.* reported an event rate of 80/1000 patient-years for hemorrhage with hospitalization with a broader definition including epistaxis, vascular access-related hemorrhage and subdural hematoma, and the study cohort included both incident and prevalent dialysis patients [2]. Of note the Canadian dialysis population may differ from international counterparts with relatively higher use of central venous catheters and warfarin all of which may impact the risk of hemorrhage [2,11].

As our rate estimates were determined in incident dialysis patients, they could serve to inform patients of their risk prior to dialysis initiation. The ability to quantitate risk of dialysis-related complications prior to dialysis initiation is increasingly important with the shift in demographics of the dialysis population to the elderly and need to balance quantity and quality of life. We further calculated the three-year cumulative incidence of hemorrhage as it may be a simple and meaningful way to communicate risk and understand disease burden from a patient perspective [25]. We found a 3-year cumulative incidence of 14.43% thus roughly 1 in 7 elderly dialysis patients will experience a hemorrhage requiring hospitalization within 3 years of dialysis initiation. This could be used in pre-dialysis discussions with patients to help them understand the risks of complications after dialysis initiation.

Among the specific subtypes of hemorrhagic events, we found upper and lower gastrointestinal hemorrhage (both well validated definitions of hemorrhage) to be the most common and roughly twenty to one hundred-fold higher in the elderly dialysis population compared to the general population [6,26,27]. Upper gastrointestinal hemorrhage has been previously reported in large US ESRD cohorts ranging from 23–57 per 1,000 person-years [10,28]. This is comparable to our rate of 20.94 per 1000 patient-years. Similarly, subarachnoid hemorrhage and intra-cerebral hemorrhage were found to occur at rates 3- to 10- fold higher in the dialysis population compared to those reported in the general population [29]. The rate of subarachnoid hemorrhage among those with ESRD was recently reported as 3-fold higher in the US compared to our study (0.74 vs. 0.33 per 1000-patient years) [29]. Differences among the rates may be attributable to a higher prevalence of associated risk factors (such as women US: 56.3% vs Canada: 42.3% and African Americans US: 25.7% vs Canada <5%) [29].

The risk of hemorrhage was stable over time, by age group and dialysis modality. The steady temporal trends have been reported in the US dialysis populations for upper gastrointestinal and subarachnoid hemorrhage, however, the comparable risk across age groups to our knowledge has not been previously reported [10,29]. The stable temporal trends in the dialysis population are in contrast to the general population where GI and SAH have been reported to be declining steadily over the last two decades [6,30]. This has been attributed to advances in prevention (acid suppressing medications) or related risk factors (hypertension treatment for subarachnoid hemorrhage) in the general population. Yet it remains unclear whether therapies are effective or treatment targets are similar or comparable in the dialysis population. The stable risk of hemorrhage with advancing age is reassuring and again provides important prognostic information for the elderly initiating dialysis. Of note, the absence of an increase in hemorrhagic events in the elderly may reflect a selection bias as elderly patients who are less likely to experience a hemorrhagic event may also be more apt to choose dialysis therapy over conservative treatments. Surprisingly despite similar hemorrhage rates, individuals ≥ 85 were much less likely to receive related endoscopic procedures (p = 0.005). Although the specific reasons for not performing endoscopic procedures were unknown, there is an increased risk of complications, often due to the bowel preparation or anesthetics, with advancing age and frailty [31]. The hemorrhagic risk appears to be similar based on dialysis modalities with the only difference detected being in gastrointestinal endoscopic procedures. It should be noted that the two cohorts are known to differ significantly in terms of demographics, co-morbidities, functionality and exposures [32]. These disparities were not accounted for in the present analysis and represent future areas on investigation.

A question of interest is the risk of hemorrhage with the use of anti-thrombotic medications [1,33–35]. We observed a large number of hemorrhagic events occurring following a recent warfarin or clopidogrel prescription. Of concern, the incidence of hemorrhage associated with clopidogrel prescription use was trending upward during the study period. To date, there remains very little evidence for efficacy of these medications in dialysis patients aside from warfarin use for a recent or recurrent thrombus [36,37]. Despite the increase in prescriptions for clopidogrel associated with hemorrhagic events over time, there was no significant increase in total hemorrhagic events. Whether this represents possible selection bias, increased use of co-interventions or declines in hemorrhagic events among patients not treated with anti-thrombotics remains unknown.

Our study has a number of strengths. Our definition of hemorrhage was stringent and limited to severe events requiring hospitalization. Previous studies included surrogate measures of bleeding (such as blood transfusion), bleeding events managed in the outpatient setting and subtypes with limited clinical impact (epistaxis) [2,3,24]. We used well validated definitions for our outcomes and excluded those lacking validation (vascular access related bleeding). We determined a three-year follow-up to ensure estimates of hemorrhage incidence would be useful for sample size calculations in future clinical trials. As CORR captures nearly all incident dialysis patients in Canada, our findings are representative and generalizable.

Our study has some limitations. We did not include traumatic or peri-operative bleeding events. Medication prescription was obtained only in cross section at the initiation of dialysis or 120 days preceding an event, and did not account for medication continuation and discontinuation over time. We lacked information on ASA use or INR control among warfarin users. We did not account for recurrent events or events in prevalent dialysis patients which would have considerably increased our risk estimates. We did not have information regarding heparin-use during the hemodialysis procedure nor did we account for changes in modality over time.

## Conclusion

Among incident dialysis patients are at high risk of hemorrhagic events requiring hospitalization. These events are stable over time, across age groups and by dialysis modality. Further studies of risk stratification for these events, and strategies to reduce these events, are needed.

## Additional file

Additional file 1: Table S1. STROBE Statement. **Table S2.** Coding definitions for demographic and co-morbid conditions. **Table S3.** Outcome Definitions. **Figure S1.** Patient selection.
