# Supplementary material for: The three-year incidence of major hemorrhage among older adults initiating chronic dialysis
Source: Can J Kidney Health Dis. 2014 Sep 2;1:21. doi: 10.1186/s40697-014-0021-x (PMC4349720; doi:10.1186/s40697-014-0021-x)
Supplement: Additional file 1: Table S1. — STROBE Statement. Table S2. Coding definitions for demographic and co-morbid conditions. Table S3. Outcome Definitions. Figure S1. Patient selection. [file 40697_2014_21_MOESM1_ESM.doc]

Additional file 1

**Table S1**: STROBE Statement

|  | Item No. | Recommendation | Reported |
| --- | --- | --- | --- |
| Title and abstract | 1 | (a) Indicate the study’s design with a commonly used term in the title or the abstract | Abstract |
|  |  | (b) Provide in the abstract an informative and balanced summary of what was done and what was found | Abstract |
| **Introduction** | | | |
| Background/  rationale | 2 | Explain the scientific background and rationale for the investigation being reported | Introduction |
| Objectives | 3 | State specific objectives, including any prespecified hypotheses | Introduction |
| **Methods** | | | |
| Study design | 4 | Present key elements of study design early in the paper | Methods – setting and design |
| Setting | 5 | Describe the setting, locations and relevant dates, including periods of recruitment, exposure, follow-up and data collection | Methods – setting and design; data sources |
| Participants | 6 | (a) Give the eligibility criteria, and the sources and methods of selection of participants. Describe methods of follow-up | Methods – Patients; Appendices |
|  |  | (b) For matched studies, give matching criteria and number of exposed and unexposed | n/a |
| Variables | 7 | Clearly define all outcomes, exposures, predictors, potential confounders, and effect modifiers. Give diagnostic criteria, if applicable | Methods – outcomes; appendices |
| Data sources/  Measurement | 8 | For each variable of interest, give sources of data and details of methods of assessment (measurement). Describe comparability of assessment methods if there is more than one group | Methods – data sources; appendices |
| Bias | 9 | Describe any efforts to address potential sources of bias | Methods – statistical analysis; Discussion |
| Study size | 10 | Explain how the study size was arrived at | n/a |
| Quantitative variables | 11 | Explain how quantitative variables were handled in the anayses. If applicable, describe which groupings were chosen and why | n/a |
| Statistical methods | 12 | (a) Describe all statistical methods, including those used to control for confounding |  |
|  |  | (b) Describe any methods used to examine subgroups and interactions | n/a |
|  |  | (c) Explain how missing data were addressed | n/a |
|  |  | (d) If applicable, explain how loss to follow-up was addressed | n/a |
|  |  | (e) Describe any sensitivity analyses |  |
| **Results** | | | |
| Participants | 13 | (a) Report numbers of individuals at each stage of study – e.g. numbers potentially eligible, examined for eligibilty, confirmed eligible, included in the study, completing follow-up, and analysed | Results; appendices |
|  |  | (b) Give reasons for non-participation at each stage | Results; appendices |
|  |  | (c) Consider use of a flow diagram | Appendix 4 |
| Descriptive data | 14 | (a) Give charchteristics of study participants (e.g. demographic, clinical, social) and information on exposures and potential confounders | Results |
|  |  | (b) Indicate number of participants with missin data for each variable of interest | n/a |
|  |  | (c) Summarise follow-up time (e.g. average and total amount) | Results |
| Outcome data | 15 | Report numbers of outcome events or summary measures over time | Results |
| Main results | 16 | (a) Give unadjusted estimates and, if applicable, confounder-adjusted estimates and their precision (e.g. 95% confidence interval). Make clear which confounders were adjusted for and why they were included | Result |
|  |  | (b) Report category boundaries when continuous variables were categorized | n/a |
|  |  | (c) If relevant, consider translating estimates of relative risk into absolute risk for a meaningful time period | n/a |
| Other analyses | 17 | Report other analyses done – e.g. analyses of subgroups and interactions, and sensitivity analyses | n/a |
| **Discussion** | | | |
| Key results | 18 | Summarise key resulst with reference to study objectives | Discussion |
| Limitations | 19 | Discuss limitations of the study, taking into account sources of potential bias or imprecision. Discuus both direction and magnitude of any potential bias | Discussion |
| Interpretation | 20 | Give a cautious overall interpretation of results considering objectives, limitations, multiplicity of analyses, results from similar studies, and other relevant evidence | Discussion |
| Gerneralisability | 21 | Discuss the gerneralisability (external validity) of the study results | Discussion |
| **Other Information** | | | |
| Funding | 22 | Give the source of funding and the role of the funders for the present study and, if applicable, for the original study on which the present article is based | Cover page |

**Table S2**: Coding definitions for demographic and co-morbid conditions

| Characteristic | Database | Codes |
| --- | --- | --- |
| Age | RPDB |  |
| Sex | RPDB |  |
| Race | CORR | Racial_Origin_Code: 01, 02, 03, 05, 08, 09, 10, 11, 98, 99 |
| Modality | CORR | Treatment_Code  Hemodialysis: 111, 112, 113, 121, 122, 123, 131, 132, 133, 211, 221, 231, 311, 312, 313, 321, 322, 323, 331, 332, 333, 413, 423, 433  In-centre HD: 111, 112, 113, 121, 122, 123, 131, 132, 133, 211, 221, 231, 311, 312, 313, 321, 322, 323, 331, 332, 333  Home HD: 413, 423, 433  Peritonal dialysis: 141, 151, 152, 241, 242, 251, 252, 443, 453 |
| Vascular Access | CORR | Dialysis_Access_Code: 1, 2, 3, 4, 5, 6, 7, 9 |
| Atrial Fibrillation | CIHI-DAD | ICD9: 4273  ICD10: I48 |
| Stroke | CIHI-DAD | ICD9: 436, 4340, 4341, 4349, 3623  ICD10: I630, I631, I632, I633, I634, I635, I638, I639, I64, H341 |
| Major Hemorrhage | CIHI-DAD | Subarachnoid Hemorrhage  ICD9: 430  ICD10: I600, I601, I602, I603, I604, I605, I606, I607, I609  Intracerebral Hemorrhage  ICD9: 431  ICD10: I61  Upper Gastrointestinal  ICD9: 5307, 5310, 5312, 5314, 5316, 5320, 5322, 5324, 5326, 5330, 5332, 5334, 5336, 5340, 5342, 5344, 5346, 5780, 5781  ICD10: I850, I9820, I983, K2210, K2211, K2212, K2214, K2216, K226, K228, K250, K252, K254, K256, K260, K262, K264, K266, K270, K272, K274, K276, K280, K282, K284, K286, K290, K2921, K2941, K2951, K2961, K2971, K2981, K2991, K3180, K31811, K3182, K6380, K920, K921  Lower Gastrointestinal  ICD9: 5693, 5789  ICD10: K5520, K625, K922 |
| Myocardial Infarction | CIHI-DAD | ICD9: 410  ICD10: I21, I22 |
| Deep Vein Thrombosis | CIHI-DAD | ICD9: 5411, 4512, 4519, 4531, 4532, 4538, 4539  ICD10: I801, I802, I803, I809, I821, I822, I828, I829 |
| Pulmonary Embolism | CIHI-DAD | ICD9: 4151  ICD10: I26 |
| Mechanical Heart Valve | CIHI-DAD  OHIP | OHIP: R772, R728, R735, R738, R863, R876  CCP: 4721, 4722, 4723, 4724, 4725, 4726, 4727, 4728, 4729  CCI: 1HS90LACF, 1HT90LACF, 1HU90LACF, 1HU90PNCF, 1HV90LACF, 1HV90LACFA, 1HV90LACFL, 1HV90LACFN, 1HV90WJCFN |

Abbreviations: RPDB, Registered Persons Database; CORR, Canadian Organ Replacement Registry; ODB, Ontario Drug Benefit; CIHI-DAD, Canadian Institute for Health Information Discharge Abstract Database; ICD, International Classification of Diseases, 9^th^ & 10^th^ revision; OHIP, Ontario Health Insurance Plan; CCP, Canadian Classification of Diagnostic, Therapeutic, and Surgical Procedures; CCI, Canadian Classification of Interventions.

**Table S3**: Outcome Definitions

| Outcome | Database | Codes | Validity |
| --- | --- | --- | --- |
| Subarachnoid Hemorrhage^1^ | CIHI-DAD | ICD9: 430  ICD10: I60.0, I60.1, I60.2, I60.3, I60.4, I60.5, I60.6, I60.7, I60.9 | ICD9  PPV: 98% (CI 90 to 99)  ICD10  PPV: 91% (CI 77 to 98) |
| Intrcerebral Hemorrhage^1^ | CIHI-DAD | ICD9: 431  ICD10: I61 | ICD9  PPV: 97% (CI 91 to 99.7)  ICD10  PPV: 98% (CI 92 to 99) |
| Upper Gastrointestinal Hemorrhage^2^ | CIHI-DAD | ICD9: 530.7, 531.0, 531.2, 531.4, 531.6, 532.0, 532.2, 532.4, 532.6, 533.0, 533.2, 533.4, 533.6, 534.0, 534.2, 534.4, 534.6, 578.0, 578.1  ICD10: I85.0, I98.20, I98.3, K22.10, K22.11, K22.12, K22.14, K22.16, K22.6, K22.8, K25.0, K25.2, K25.4, K25.6, K26.0, K26.2, K26.4, K26.6, K27.0, K27.2, K27.4, K27.6, K28.0, K28.2, K28.4, K28.6, K29.0, K29.21, K29.41, K29.51, K29.61, K29.71, K29.81, K29.91, K31.80, K31.811, K31.82, K63.80, K92.0, K92.1 | Sensitivity: 94% (CI 91 to 96)  Specificity: 83% (CI 78 to 87) |
| Lower Gastrointestinal Hemorrhage^2^ |  | ICD9: 569.3, 578.9  ICD10: K55.20, K62.5, K92.2 | Sensitivity: 94% (CI 91 to 96)  Specificity: 83% (CI 78 to 87) |
| Digestive System Endoscopy | OHIP | Feecode: Z515, Z399, Z400, E696, E702, E690, E795, E770, E692, E698, E703, E799, E695, E797, E798, E629,Z527, Z547,  Z528, E674, E675, Z560, Z749, E629, Z584, Z512, E747, Z514, Z555, E740, E741, E747, E705, Z580, Z497, Z496, Z535, Z536, Z592, E746, E641, E797 |  |

Abbreviations: CIHI-DAD, Canadian Institute for Health Information Discharge Abstract Database; OHIP, Ontario Health Insurance Plan; ICD, International Classification of Diseases; RPDB, Registered Persons Database.

*Codes may appear at any time during a patient’s admission (and may not necessarily be their most responsible diagnosis) unless otherwise specified.

**Figure S1**: Patient selection

**Patients on chronic dialysis in Ontario, 1998 to 2008**

**n= 25,258**

**Excluded:**

**26 Patients with invalid provincial health card number or missing age or sex (data cleaning step)**

**14 Patients who died prior to the start of chronic dialysis (data cleaning step)**

**12,366 Patients <66 years of age at the start of chronic dialysis**

**171 Patients with a history of a kidney transplant prior to the start of chronic dialysis**

**470 Patients with a history of chronic dialysis >180 days prior to their first chronic dialysis treatment date**

**1,013 Patients who died prior to hospital discharge, if dialysis was initiated during a hospital admission**

**25 Patients whose hospital discharge date was beyond December 31, 2008, (the last date of patient accrual)**

**36,489 Total**

**Patients included in the analysis**

**n= 11,173**
